# Association of sex hormone-binding globulin with nonalcoholic fatty liver disease in Chinese adults

**DOI:** 10.1186/s12986-018-0313-8

**Published:** 2018-11-08

**Authors:** Jing Luo, Qian Chen, Tianran Shen, Xu Wang, Wanjun Fang, Xiaocai Wu, Zenan Yuan, Gengdong Chen, Wenhua Ling, Yuming Chen

**Affiliations:** 1 Department of Nutrition, School of Public Health, Sun Yat-sen University (Northern Campus), Guangzhou, 510080 China; 2 Guangdong Provincial Key Laboratory of Food, Nutrition and Health, Guangzhou, 510080 China; 3 Department of Medical Statistics and Epidemiology, School of Public Health, Sun Yat-sen University (Northern Campus), Guangzhou, 510080 China; 4 Department of Hepatic Surgery,The Third Affiliated Hospital, Sun Yat-sen University, Guangzhou, 510630 China; 5 Department of Cardiology, Sun Yat-sen Memorial Hospital, Sun Yat-sen University, Guangzhou, 510120 China

**Keywords:** Sex hormone-binding globulin, Nonalcoholic fatty liver disease, Hepatic steatosis, Intrahepatic triglyceride

## Abstract

**Background:**

Sex hormone-binding globulin (SHBG), a glycoprotein synthesized by hepatocytes, has been linked to insulin resistance and hepatic lipid metabolism and is suggested to be associated with nonalcoholic fatty liver disease (NAFLD). This study aimed to investigate the association of SHBG with NAFLD in Chinese adults.

**Methods:**

We conducted a community-based, cross-sectional study in China involving 2912 participants aged 40–75 years old. All participants underwent detection for hepatic fat infiltration by ultrasound in addition to providing complete medical history and undergoing physical and blood biochemical examinations. The association of serum SHBG with the presence of NAFLD was reported by adjusted odds ratio after applying logistic regression models. To further explore the relationship between SHBG and NAFLD, mRNA expression of SHBG and hepatocyte nuclear factor 4-α (HNF4α), as well as intrahepatic triglycerides, were determined from the liver tissues of 32 subjects with different degrees of steatosis.

**Results:**

Serum SHBG levels in patients with NAFLD (median, 43.8 nmol/L; interquartile range, 33.4–56.8 nmol/L) were significantly lower than those in non-NAFLD subjects (median, 63.4 nmol/L; interquartile range, 47.6–83.1 nmol/L). Serum SHBG levels were inversely correlated with WHR, trunk fat percentage, glucose, HOMA-IR, TG, UA and DHEAS, and were positively correlated with HDL-C levels (all *p* <  0.001). Logistic regression analysis indicated that serum SHBG levels were negatively associated with the presence of NAFLD in all subjects, as well as the subgroups stratified by sex, BMI and HOMA-IR (all *p* <  0.05). In human liver tissues, SHBG and HNF4α mRNA expression decreased along with the elevated grade of hepatic steatosis. Both SHBG and HNF4α mRNA expression levels were negatively correlated with intrahepatic triglycerides.

**Conclusions:**

These results demonstrate that SHBG levels were negatively associated with the presence of NAFLD in middle-aged and elderly Chinese adults.

**Electronic supplementary material:**

The online version of this article (10.1186/s12986-018-0313-8) contains supplementary material, which is available to authorized users.

## Introduction

Nonalcoholic fatty liver disease (NAFLD) is now acknowledged as a global public health issue [1]. It is estimated that the global prevalence of NAFLD is 25.24% [2]. In China, 20.09% of the general adult population is potentially influenced by NAFLD [3]. NAFLD has become one of the most common causes of chronic liver disease. It is regarded to be the hepatic manifestation of metabolic syndrome or insulin resistance [4], increasing in parallel with the rise in cardiovascular or cerebrovascular diseases and type 2 diabetes mellitus [5, 6]. The spectrum of NAFLD ranges from simple liver steatosis to steatohepatitis, which may progress to advanced fibrosis, cirrhosis or hepatocellular carcinoma [7]. Most patients with NAFLD are asymptomatic or complain about nonspecific symptoms (for example, fatigue, right upper quadrant discomfort or sleep disorder), which makes it difficult to screen for NAFLD [8].

As our understanding in the occurrence and development of NAFLD continues to evolve, sex hormone-binding globulin (SHBG) has been suggested to be associated with NAFLD. SHBG is a 90–100 KDa homodimeric glycoprotein, mainly secreted by hepatocytes [9]. It has traditionally been considered to function as a transporter of sex steroids, controlling circulating free hormone concentrations [10]. Numerous publications have proposed that low levels of SHBG are strongly associated with the features of metabolic syndrome [11–13] and may play an important role in modifying the risk of insulin resistance and its outcomes [14–16]. An inverse association between SHBG and the risk of NAFLD was observed in diabetic patients and women with polycystic ovarian syndrome [17–20]. Wang N et al. also demonstrated that lower levels of SHBG were associated with the presence of NAFLD in Chinese men and menopausal women [21]. In contrast to the above findings, the results of a population-based study involving a survey of 1882 men showed that an increase in SHBG levels occurred in men with NAFLD [22]. Though a meta-analysis of associations between testosterone and SHBG and NAFLD which extracted data from 16 observational studies claimed that SHBG was lower in subjects with NAFLD compared to those without NAFLD [23], a significant heterogeneity was observed in the study (I^2^ = 99% in men, I^2^ = 97% in women, both *p* for heterogeneity < 0.01), which reduced the credibility of the conclusion. Therefore, it is still difficult to draw firm conclusions about the relationship between SHBG and NAFLD for people from the general population, especially when we discuss the problem in the Chinese population.

Nuclear receptor hepatocyte nuclear factor 4-α (HNF4α) activates the promoters of multiple genes expressed in hepatocytes that play a role in lipoprotein metabolism [24]. The proximal promoter of the SHBG gene contains an HNF4α binding site, and overexpression of transcription factor HNF4α in HepG2 cells can stimulate transcription of the SHBG promoter [25]. Several studies found that monosaccharide and lipid, as well as other factors, could regulate the SHBG expression via changes in HNF4α gene expression [26–28]. Among them, liver lipids were considered to be a crucial factor in regulating HNF4α-SHBG. Adiponectin treatment of HepG2 cells activated AMPK, which decreased the hepatic lipid content, then increased HNF4α levels and upregulated SHBG expression [28]. SHBG mRNA is not naturally expressed in rodent liver; therefore, to date, research on SHBG gene expression using in vivo models is limited, and studies concerning SHBG expression in humans are rarely found.

Accordingly, we conducted a large, community-based, cross-sectional study to evaluate the association between SHBG and the prevalence of NAFLD in middle-aged and elderly Chinese adults. Moreover, we furthered our study by including liver biopsy samples in order to improve our understanding of the relevance between SHBG and the risk of NAFLD in humans.

## Methods

### Participants

The study was based on the Guangzhou Nutrition and Health Study (GNHS), a community-based prospective cohort study aimed to investigate the potential association between dietary nutrition intake, genetic factors and noncommunicable chronic diseases, along with their interactions. Between July 2008 and June 2010, 3169 participants aged 40–75 years old who had at least a 5-year continuous residence in Guangzhou were initially recruited into this study through advertisements, health talks, and referrals from local community centres. Of these participants, 2520 completed the initial survey, while 649 subjects dropped out due to refusal, loss of contact, emigration or death between April 2011 and May 2013. Additional 869 participants were recruited between March 2013 and October 2013 using the same methods. Information on demographic characteristics were collected in both 2008–2010 and 2011–2013. Fasting venous blood samples were obtained and B-scan ultrasonography was measured during the 2011–2013 survey.

**Fig. 1:**
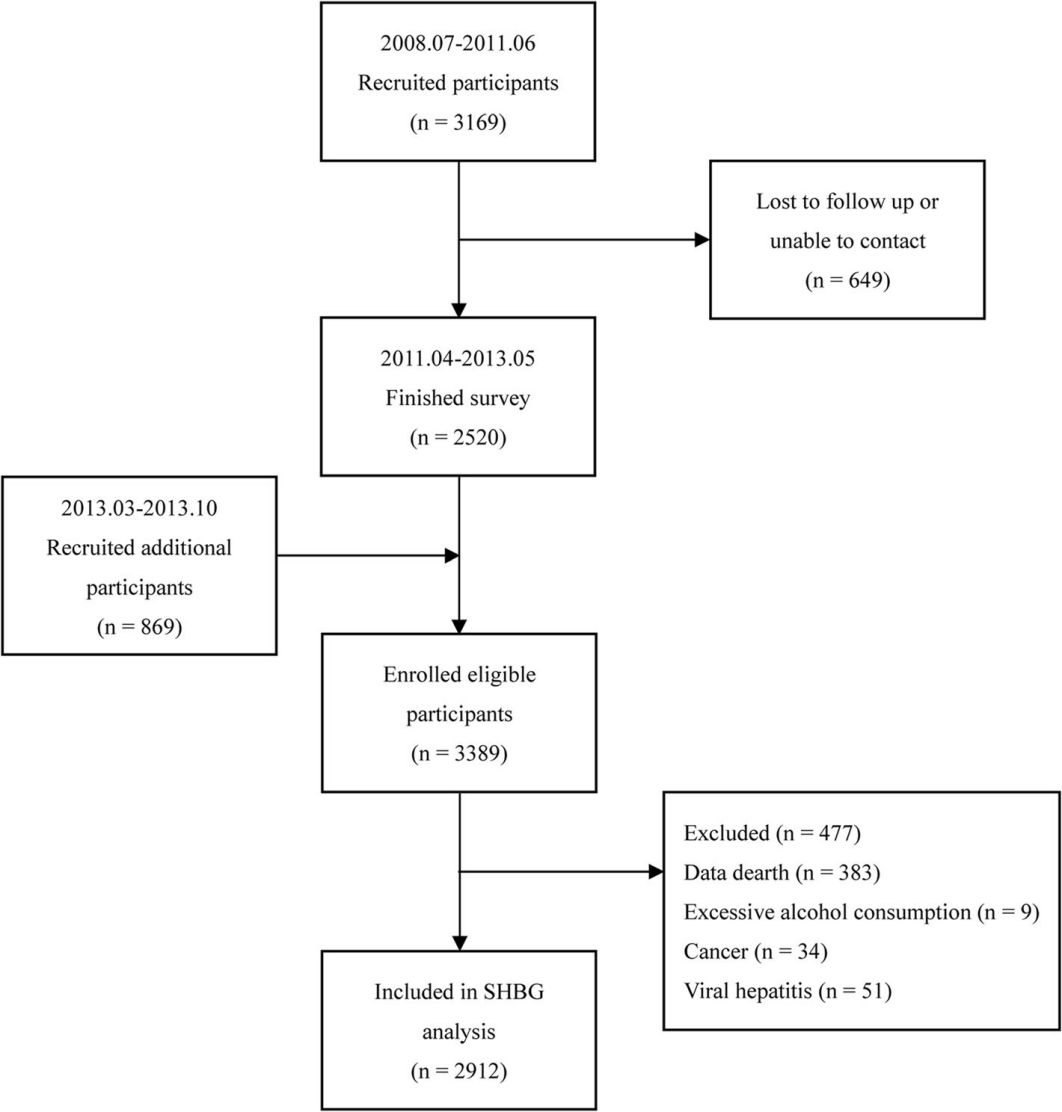
Flow-chart of the recruitment of community participants

Participants who had missing blood biochemical index values or were without abdominal ultrasonographic results were excluded, and 3006 participants remained available for analysis. Furthermore, subjects with any of the following conditions were excluded: excessive alcohol consumption (≥ 140 g/wk. for males or ≥ 70 g/wk. for females) [29]; viral hepatitis [29]; other causes of liver disease (autoimmune, genetic, etc.); haemochromatosis [30]; biliary obstructive diseases; the presence of severe medical diseases (cancer, stroke, heart failure, etc.); current treatment with systemic corticosteroids; and pregnancy [30]. In the end, 2912 participants were enrolled in this analysis (the flow-chart of the study is presented in Fig. 1).

### Data collection

Each participant underwent a comprehensive physical examination, routine biochemical analysis of fasting blood, hepatitis virus test and B-scan ultrasonography. Trained researchers administered face-to-face interviews to collect information on participants’ demographic characteristics, lifestyle and habits (e.g., smoking, alcohol drinking and physical activity) and history of chronic diseases through standardized questionnaires. Subjects who drank alcohol once a week or smoked at least one cigarette per day for at least 6 months were identified as drinkers or smokers. Body mass index (BMI) was calculated as weight (kg) divided by the square of height (m^2^), and subjects with values greater than 24 kg/m^2^ were identified as overweight by the Chinese standard proposed by the Working Group on Obesity in China [31]. The metabolic equivalent (MET) intensity was calculated to estimate daily physical activity using a 24-h physical activity questionnaire [32]. The fat mass of the trunk region was quantified using dual-energy X-ray absorptiometry scans (Discovery W; Hologic Inc., Waltham, MA, USA). The trunk region was defined as the area of the trunk between an upper horizontal boundary below the chin and a lower boundary above the oblique lines through each hip joint [33].

### Clinical diagnosis and rating for NAFLD

Abdominal ultrasonography was used to diagnose NAFLD on a Doppler sonography machine (Sonoscape SSI-5500, Shenzhen, China) with a 3.5 MHz probe. Ultrasound scanning for all participants was performed by two experienced technicians in the same clinical setting who were blinded to the clinical and laboratory data. The diagnosis of NAFLD was conducted following standard criteria issued by the Fatty Liver Disease Study Group of the Chinese Liver Disease Association [29, 34], and hepatic steatosis was semi-quantitatively rated as absent, mild, moderate or severe on the basis of ultrasonographic phenomenon including hepatorenal echo contrast, liver brightness, deep attenuation and vessel structure blurring. Between-operator reliability evaluation for ultrasound scanning was determined in 100 participants, showing a very good precision (Spearman’s *r* = 0.911, kappa = 0.875, total agreement = 93%, *p* <  0.001). Validity evaluation was assessed in 34 participants who further underwent computed tomography (CT), with the technicians blinded to the ultrasound results, a good agreement was observed (Spearman’s *r* = 0.905, kappa = 0.691, total agreement = 85%, *p* <  0.001).

### Human liver tissue

Thirty-two patients undergoing hepatic resection as treatment for hepatic benign tumour in The Third Affiliated Hospital of Sun Yat-sen University were recruited for this study, which was approved by Sun Yat-sen University’s School of Public Health Ethics Committee. Following informed consent, the patients’ medical history was reviewed, and biochemical parameters were collected. Subjects with excessive alcohol consumption and other liver diseases, such as hepatitis B, hepatitis C and cirrhosis were excluded. After surgery to remove a liver specimen, tumour was removed for clinical analysis, and adjacent normal liver was stored in liquid nitrogen for subsequent analysis.

The degree of steatosis in sections of the liver specimens were assessed by two pathologists in a blinded fashion. The histological features of the liver were graded according to a histological scoring system for NAFLD [35].

### Biochemical measurements

All venous blood samples were obtained after overnight fasting. The serum was separated into several aliquots and stored at − 80 °C within 2 h. Serum glucose, triglycerides (TG), total cholesterol (TC), high-density lipoprotein cholesterol (HDL-C), low-density lipoprotein cholesterol (LDL-C), aspartate aminotransferase (AST), alanine aminotransferase (ALT) and uric acid (UA) were determined enzymatically on a Hitachi 7600–010 automated analyser (Hitachi, Tokyo, Japan). Insulin resistance was evaluated using the homeostasis model assessment (HOMA) index [fasting glucose (mmol/L) × insulin (μU/mL) / 22.5]. Serum SHBG, testosterone (Testo) and dehydroepiandrosterone sulphate (DHEAS) were measured by chemiluminescent enzyme immunometric assay (CLIA) using kits obtained from Abbott Corporation (Chicago, IL, USA) with an Abbott ARCHITECT I2000 automated analyser. The minimum detectable dose of SHBG was 2 nmol/L, and the intra- and inter-assay coefficients of variation for SHBG were 4.1% and 5.8%, respectively.

### RNA extraction and quantitative real-time PCR

Total RNA from human liver tissues was isolated using TRIzol reagent (Invitrogen, Carlsbad, CA, USA), and reverse transcription was performed with a PrimeScript RT Reagent Kit (TaKaRa, Tokyo, Japan) according to the manufacturer’s instructions. The mRNA expression was quantified in a 96-well plate using a 7500 Real-Time PCR System (Applied Biosystems, Inc., Foster City, CA, USA) with SYBR Green Master Mix (TaKaRa, Tokyo, Japan). The relative mRNA expression levels were determined using the 2^-ΔΔCt^ method with GAPDH as an internal control for normalization. The sequences of forward and reverse primers used in the current study were: human SHBG, 5’-GCCCAGGACAAGAGCCTATC-3′ and 5’-CCTTAGGGTTGGTATCCCCATAA-3′; human HNF4α, 5’-AACACCTCAACAAGGGCACTC-3′ and 5’-CCCCACTTGAAACG-GTTCCT-3′; human GAPDH, 5’-GGAGCGAGATCCCTCCAAAAT-3′ and 5’-GGCTGTTGTCATACTTCTCATGG -3′.

### Analysis of liver TG content

Human liver samples were immediately shock-frozen and grinded in liquid nitrogen to measure the intrahepatic TG concentration, which was determined enzymatically after extraction using a commercial kit (Applygen Technologies Inc., Beijing, China) according to the manufacturer’s instructions.

### Statistical analysis

Data were presented as means ± standard deviation or median (interquartile range) for continuous variables and as frequencies (percentages) for categorical variables. Differences between groups of continuous variables were compared using either independent samples t-test or Mann-Whitney U test. Chi-square test was used to compare categorical variables between groups. Spearman’s correlation analysis and multiple linear regression were used to estimate the association between serum SHBG levels and several relevant metabolic factors. Logistic regression analysis was used to estimate the odds ratios (ORs) and 95% confidence intervals (CIs) for the risk of NAFLD with increasing quartiles of serum SHBG levels, using the lowest quartile as the reference group. Logistic regression analyses were performed separately for different sexes, different BMI and HOMA-IR status, and the interactions between serum SHBG concentrations and BMI levels as well as HOMA-IR were tested. We adjusted for age, sex, postmenopausal status (for females) and household income in Model 1. To investigate the independent associations, we further adjusted for waist-to-hip ratio (WHR), trunk fat percentage, current smoking and drinking, physical activity (MET), hypertension and diabetes in Model 2, plus serum glucose, HOMA-IR, ALT, triglycerides, HDL-C, UA, testosterone and DHEAS levels in Model 3. Analysis of covariance (ANCOVA) was used to identify differences between groups, and the variables were adjusted as in Model 3. Kruskal-Wallis test was used as appropriate for comparisons of mRNA expression in liver tissue between groups, and multiple testing was corrected using Bonferroni correction. Correlations of hepatic TG with serum SHBG levels, SHBG mRNA expression and HNF4α mRNA expression in liver tissue were examined using Pearson’s correlation analysis. A two-tailed *p*-value < 0.05 was considered statistically significant. All of the statistical procedures were performed using SPSS Statistics software (version 22.0, SPSS Inc., Chicago, IL, USA).

## Results

### Characteristics of participants

The characteristics of the participants are presented in Additional file 1: Table S1. The study involved 2912 participants, of whom 2009 were women. NAFLD group presented with higher BMI levels than non-NAFLD group, as well as a higher WHR and trunk fat percentage. In addition, participants with NAFLD had higher serum levels of ALT, glucose, HOMA-IR, TG and UA, and lower physical activity and HDL-C levels than non-NAFLD subjects in both females and males (all *p* <  0.05).

### Serum SHBG levels in non-NAFLD and NAFLD subjects

**Fig. 2:**
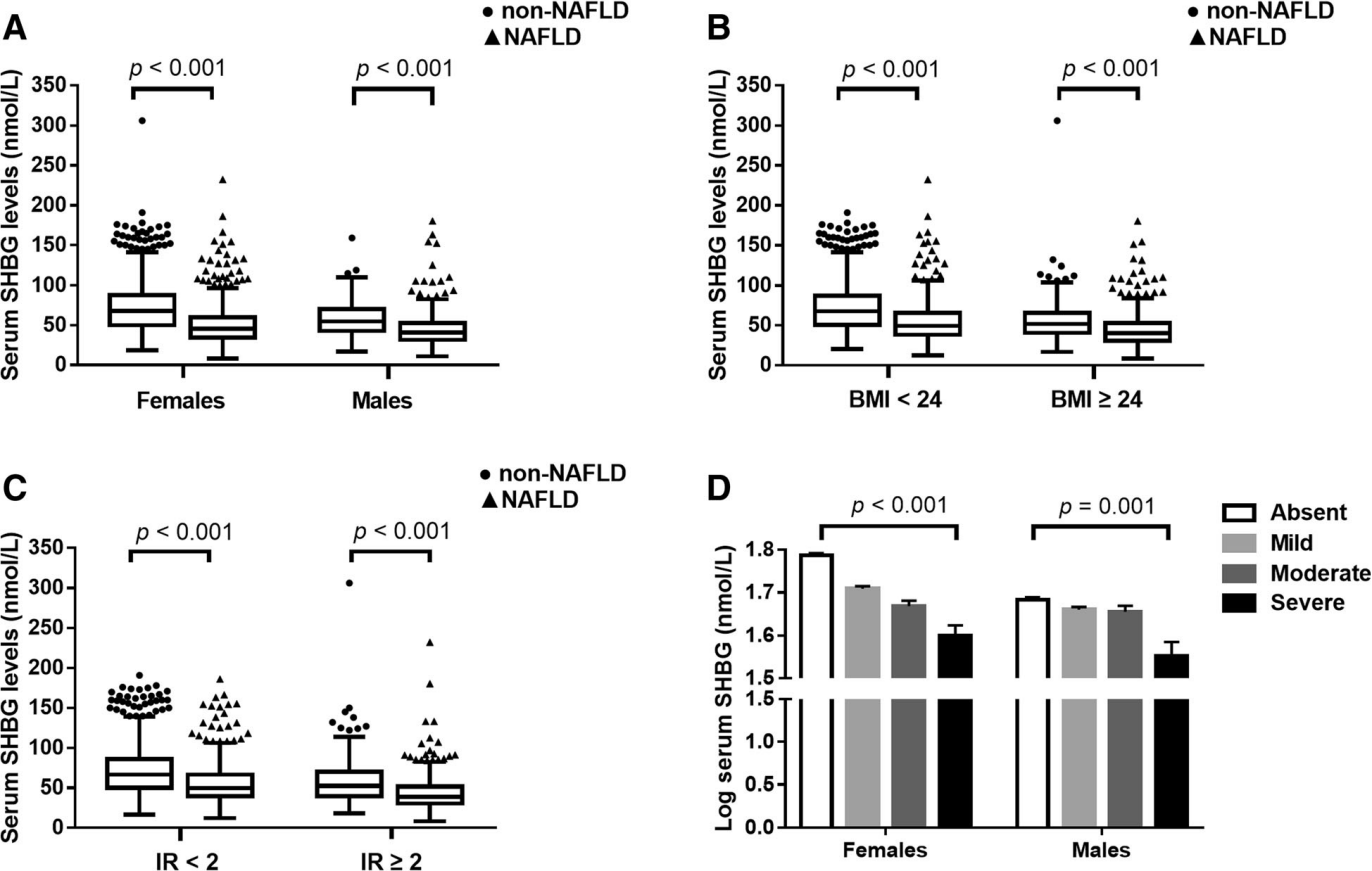
Serum SHBG concentrations (nmol/L) in non-NAFLD subjects and NAFLD patients. Serum levels of SHBG in individuals with or without NAFLD stratified by sex (**a**), BMI (**b**) and HOMA-IR (**c**). The box plots display median values and 25th and 75th percentiles; the whiskers represent 25th percentiles - 1.5 * interquartile range and 75th percentiles + 1.5 * interquartile range. (**d**) Multivariable adjusted means (SEM) of SHBG (log-transformed) according to the severity of NAFLD. After being logarithmically transformed, SHBG was adjusted for age, postmenopausal status (for females), household income, WHR, trunk fat, current smoking and drinking, physical activity (MET), hypertension and diabetes, serum glucose, insulin resistance (HOMA-IR), TG, HDL-C, ALT, UA, testosterone and DHEAS levels using ANCOVA

In this study, the non-NAFLD participants had much higher SHBG concentrations (median, 63.4 nmol/L; interquartile range, 47.6–83.1 nmol/L) than the NAFLD participants (median, 43.8 nmol/L; interquartile range, 33.4–56.8 nmol/L) (*p* <  0.001). Additionally, as shown in Fig. 2a-c, serum SHBG concentrations exhibited significant differences between non-NAFLD subjects and NAFLD patients regardless of sex (median, 67.9 nmol/L versus 45.6 nmol/L in females; 54.8 nmol/L versus 40.6 nmol/L in males), BMI (median, 67.6 nmol/L versus 49.0 nmol/L in subjects with BMI < 24 kg/m^2^; 51.7 nmol/L versus 40.2 nmol/L in subjects with BMI ≥ 24 kg/m^2^) and HOMA-IR status (median, 66.3 nmol/L versus 49.7 nmol/L in subjects with HOMA-IR < 2; 52.3 nmol/L versus 38.8 nmol/L in subjects with HOMA-IR ≥ 2) (all *p* <  0.001).

As shown in Fig. 2d, we observed that serum SHBG levels (log-transformed) decreased gradually with increased severity of hepatic steatosis in both females and males after adjustments for the variables [age, postmenopausal status (for females), household income, WHR, trunk fat, current smoking and drinking, physical activity, hypertension and diabetes, serum glucose, insulin resistance, TG, HDL-C, ALT, UA, testosterone and DHEAS levels] (both *p* -trend < 0.05).

### Association between serum SHBG levels and metabolic risk factors

We next investigated the correlation of serum SHBG levels with a cluster of anthropometric parameters and biochemical indices. The results are shown in Table 1. The analysis demonstrated negative correlations of serum SHBG with sex, BMI, WHR, trunk fat percentage and several biochemical parameters, including ALT, glucose, HOMA-IR, TG, TC, UA, testosterone and DHEAS (all *p* <  0.001). A positive correlation of SHBG with HDL-C levels was observed (*p* <  0.001). After adjustment for age, sex and BMI, correlation of serum SHBG levels with WHR, trunk fat percentage, glucose, HOMA-IR, TG, UA, DHEAS and HDL-C still existed robustly (all *p* <  0.001). We further conducted multiple regression analysis to examine the relationships that involved the above parameters. As shown in Additional file 2: Table S2, serum SHBG levels (log-transformed) were treated as a dependent variable. In all subjects, BMI, WHR, trunk fat, ALT, fasting glucose, HOMA-IR, TG, HDL-C, cholesterol, LDL-C and UA were all chosen as independent variables before and after adjusting for age, sex, current smoking and drinking, physical activity, hypertension, diabetes (except for cholesterol and LDL-C, all *p* <  0.001).

**Table 1:** Univariate and multivariate-adjusted Spearman correlation coefficients of SHBG and metabolic risk factors

Variables	SHBG		SHBG (age, sex and BMI-adjusted)	
	*r*	*p*	*r*	*p*
Age (years)	0.027	0.144	–	–
Sex	−0.177	< 0.001	–	–
BMI (kg/m^2^)	−0.455	< 0.001	–	–
WHR	−0.327	< 0.001	−0.186	< 0.001
Trunk fat percentage (%)	−0.205	< 0.001	−0.232	< 0.001
ALT (U/L)	−0.206	< 0.001	−0.029	0.117
Glucose (mmol/L)	−0.208	< 0.001	−0.114	< 0.001
HOMA-IR	−0.467	< 0.001	−0.177	< 0.001
Triglyceride (mmol/L)	−0.386	< 0.001	−0.192	< 0.001
Cholesterol (mmol/L)	−0.067	< 0.001	−0.015	0.406
HDL-C (mmol/L)	0.390	< 0.001	0.269	< 0.001
LDL-C (mmol/L)	0.023	0.215	−0.026	0.168
UA (μmol/L)	−0.331	< 0.001	−0.206	< 0.001
Testosterone (ng/dL)	−0.105	< 0.001	0.235	< 0.001
DHEAS (μg/dL)	−0.216	< 0.001	−0.122	< 0.001

The age, sex and BMI-adjusted associations between serum SHBG levels and several relevant factors were estimated by partial correlation

*SHBG* sex hormone-binding globulin; *BMI* body mass index; *WHR* waist-to-hip ratio; *ALT* alanine aminotransferase; *HOMA-IR* homeostasis model assessment of insulin resistance; *HDL-C* high-density lipoprotein cholesterol; *LDL-C* low-density lipoprotein cholesterol; *UA* uric acid; *DHEAS* dehydroepiandrosterone sulphate

### Association of serum SHBG levels with NAFLD

To further explore the associations of serum SHBG levels with NAFLD, we employed logistic regression models to examine the sex-specific associations of serum SHBG quartiles with the odds of the presence of NAFLD. As shown in Table 2, We found that higher serum SHBG quartiles were associated with lower odds of NAFLD in all subjects (OR for the highest quartile versus the lowest quartile, 0.09; 95% CI, 0.07–0.11; *p* <  0.001) after adjusting for age, sex and household income in binary logistic regression. In the following analysis stratified by sex, the initial association remained the same in both females and males (all *p* <  0.001). Furthermore, these associations still maintained robustness after adjustment for physical and metabolic parameters in Models 2 and 3, respectively. The adjusted ORs of NAFLD in Model 3 for the highest (versus the lowest) quartile were 0.24 (95% CI, 0.18–0.32) in all participants, 0.19 (95% CI, 0.13–0.27) in females and 0.42 (95% CI, 0.23–0.77) in males (all *p* <  0.05). Otherwise, we still found significant inverse associations between SHBG and NAFLD regardless of BMI (*p* for interaction = 0.150; for subjects with BMI < 24 kg/m^2^, adjusted OR in Model 3, 0.31, 95% CI, 0.21–0.45, *p* <  0.001; for subjects with BMI ≥ 24 kg/m^2^, adjusted OR in Model 3, 0.31, 95% CI, 0.19–0.51, *p* <  0.001) and HOMA-IR status (*p* for interaction = 0.021; for subjects with HOMA-IR < 2, adjusted OR in Model 3, 0.34, 95% CI, 0.24–0.49, *p* <  0.001; for subjects with HOMA-IR ≥ 2, adjusted OR in Model 3, 0.22, 95% CI, 0.13–0.36, *p* <  0.001) (Table 3).

**Table 2:** Adjusted odds ratios (95% confidence intervals) of NAFLD according to sex and serum SHBG quartiles using adjusted logistic regression

	SHBG (nmol/L)				*p* for trend
	Quartile 1	Quartile 2	Quartile 3	Quartile 4	
Total (*N* = 2912)	< 38.70	38.70–52.45	52.46–71.3	> 71.3	
Model 1	1.00	0.38 (0.30–0.48)	0.21 (0.16–0.26)	0.09 (0.07–0.11)	< 0.001
Model 2	1.00	0.46 (0.36–0.59)	0.30 (0.23–0.38)	0.18 (0.13–0.23)	< 0.001
Model 3	1.00	0.54 (0.42–0.70)	0.38 (0.29–0.50)	0.24 (0.18–0.32)	< 0.001
Females (*N* = 2009)	< 40.65	40.65–55.70	55.71–75.80	> 75.80	
Model 1	1.00	0.34 (0.25–0.45)	0.18 (0.14–0.24)	0.07 (0.05–0.09)	< 0.001
Model 2	1.00	0.40 (0.30–0.54)	0.24 (0.18–0.33)	0.13 (0.09–0.18)	< 0.001
Model 3	1.00	0.49 (0.36–0.67)	0.33 (0.24–0.45)	0.19 (0.13–0.27)	< 0.001
Males (*N* = 903)	< 35.50	35.50–47.10	47.11–61.00	> 61.00	
Model 1	1.00	0.67 (0.45–1.00)	0.31 (0.21–0.46)	0.13 (0.09–0.21)	< 0.001
Model 2	1.00	0.83 (0.53–1.29)	0.47 (0.30–0.73)	0.35 (0.22–0.57)	< 0.001
Model 3	1.00	0.95 (0.59–1.53)	0.57 (0.35–0.95)	0.42 (0.23–0.77)	0.001

Model 1: Adjusted for age, sex, postmenopausal status (for females) and household income

Model 2: Adjusted for the variables in Model 1 plus WHR, trunk fat, current smoking and drinking, physical activity (MET), hypertension and diabetes

Model 3: Adjusted for the variables in Model 2 plus serum glucose, insulin resistance (HOMA-IR), TG, HDL-C, ALT, UA, testosterone and DHEAS levels

NAFLD, nonalcoholic fatty liver disease; SHBG, sex hormone-binding globulin

**Table 3:** Adjusted odds ratios (95% confidence intervals) of NAFLD according to BMI and HOMA-IR status and serum SHBG quartiles using adjusted logistic regression

	SHBG (nmol/L)				*p* for trend
	Quartile 1	Quartile 2	Quartile 3	Quartile 4	
BMI < 24 kg/m^2^ (*N* = 1696)	< 45.03	45.03–60.90	60.91–80.98	> 80.98	
Model 1	1.00	0.50 (0.38–0.66)	0.23 (0.17–0.31)	0.15 (0.11–0.21)	< 0.001
Model 2	1.00	0.56 (0.42–0.75)	0.29 (0.21–0.40)	0.24 (0.17–0.34)	< 0.001
Model 3	1.00	0.60 (0.46–0.84)	0.35 (0.25–0.48)	0.31 (0.21–0.45)	< 0.001
BMI ≥ 24 kg/m^2^ (*N* = 1216)	< 32.70	32.70–43.10	43.11–56.30	> 56.30	
Model 1	1.00	0.53 (0.34–0.83)	0.33 (0.21–0.51)	0.17 (0.11–0.27)	< 0.001
Model 2	1.00	0.57 (0.36–0.89)	0.37 (0.24–0.57)	0.21 (0.14–0.32)	< 0.001
Model 3	1.00	0.70 (0.44–1.12)	0.49 (0.30–0.78)	0.31 (0.19–0.51)	< 0.001
*p* for interaction	–	–	–	–	0.150
HOMA-IR < 2 (*N* = 1780)	< 45.20	45.20–60.10	60.11–79.38	> 79.38	
Model 1	1.00	0.55 (0.42–0.72)	0.29 (0.22–0.39)	0.17 (0.13–0.23)	< 0.001
Model 2	1.00	0.64 (0.49–0.85)	0.40 (0.29–0.54)	0.32 (0.23–0.44)	< 0.001
Model 3	1.00	0.67 (0.50–0.89)	0.43 (0.31–0.59)	0.34 (0.24–0.49)	< 0.001
HOMA-IR ≥ 2 (*N* = 1132)	< 31.50	31.50–41.60	41.61–55.68	> 55.68	
Model 1	1.00	0.76 (0.47–1.21)	0.41 (0.27–0.64)	0.16 (0.10–0.25)	< 0.001
Model 2	1.00	0.84 (0.52–1.36)	0.46 (0.29–0.73)	0.21 (0.14–0.33)	< 0.001
Model 3	1.00	0.83 (0.50–1.36)	0.43 (0.27–0.70)	0.22 (0.13–0.36)	< 0.001
*p* for interaction	–	–	–	–	0.021

Model 1: Adjusted for age, sex and household income

Model 2: Adjusted for the variables in Model 1 plus WHR, trunk fat, current smoking and drinking, physical activity (MET), hypertension and diabetes

Model 3: Adjusted for the variables in Model 2 plus serum glucose, insulin resistance (HOMA-IR), TG, HDL-C, ALT, UA, testosterone and DHEAS levels

*NAFLD* nonalcoholic fatty liver disease; *SHBG* sex hormone-binding globulin; *BMI* body mass index; *HOMA-IR* homeostasis model assessment of insulin resistance

### SHBG expression in liver tissue of NAFLD patients

**Fig. 3:**
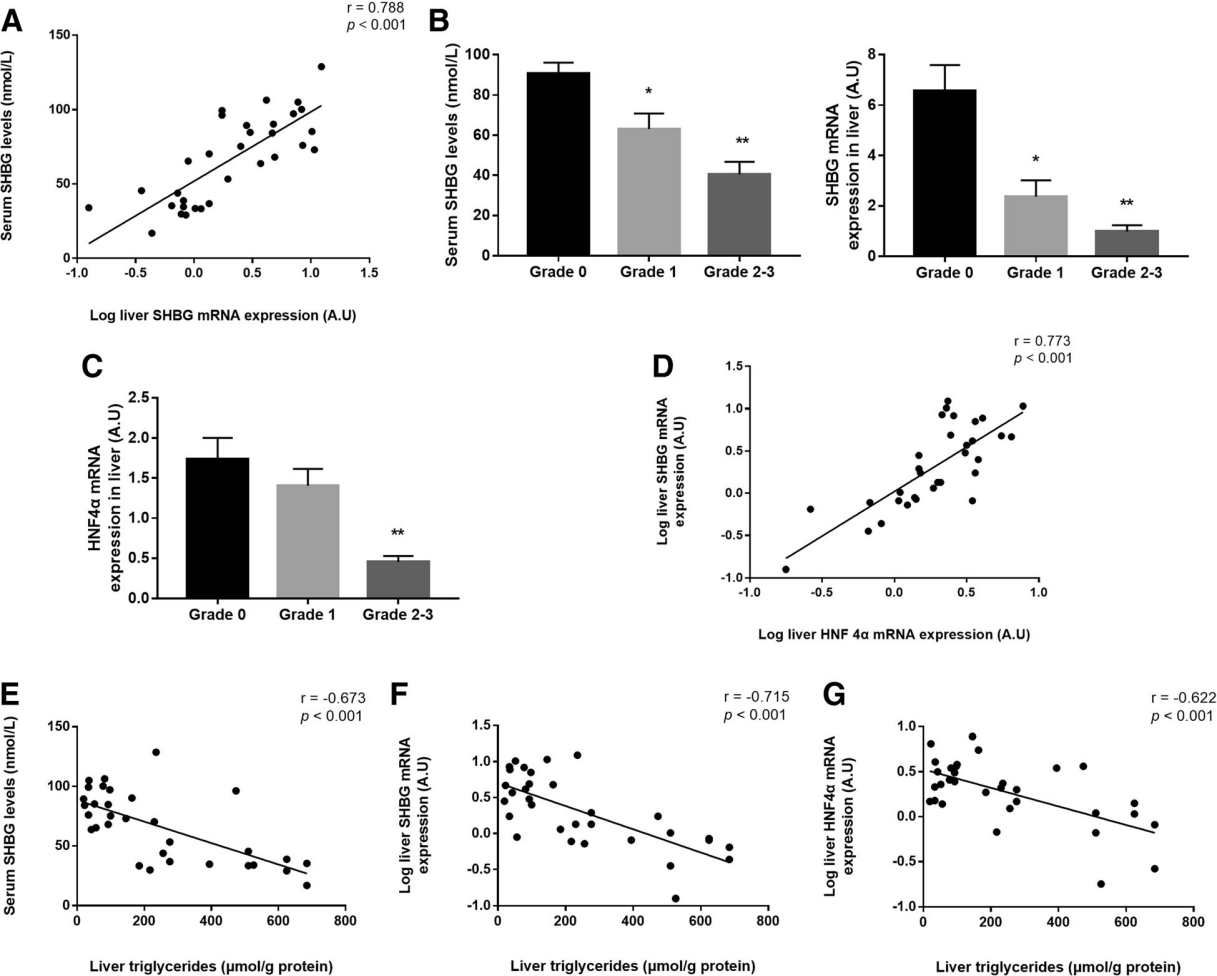
SHBG and HNF4α mRNA expression in fatty liver tissues. (**a**) Correlation analysis of the relationship between SHBG (log-transformed) and serum SHBG levels. (**b**-**c**) Serum SHBG levels and SHBG and HNF4α mRNA expression in fatty liver tissue [mean (SEM)] between Grade 0 (n = 12), Grade 1 (*n* = 10) and Grade 2–3 (*n* = 10) versus Grade 0 steatosis, *p < 0.05 **p < 0.01. (D) Correlation of SHBG mRNA expression (log-transformed) with HNF4αmRNA expression (log-transformed). (**e**-**g**) Correlations of hepatic TG with serum SHBG levels, SHBG mRNA expression (log-transformed) and HNF4α mRNA expression (log-transformed)

We next examined the expression of SHBG mRNA in liver tissue obtained from 32 patients. The characteristics of the participants are presented in Additional file 2: Table S3. A positive correlation was found between the expression of SHBG mRNA in liver and the level of serum SHBG (*r* = 0.788, *p* <  0.001; Fig. 3a). The liver SHBG mRNA expression and serum SHBG level of Grade 1 steatosis was respectively 2.78- and 1.43-fold lower than that in tissues of Grade 0 (*p* <  0.05). Due to the small sample sizes in Grades 2 and 3, the two groups were combined for analysis. In Grade 2–3 liver tissues, the SHBG mRNA expression and serum SHBG level was respectively 6.72- and 2.23-fold lower than that in tissues of Grade 0 (*p* <  0.001) (Fig. 3b). As shown in Fig. 3c, HNF4α mRNA expression in Grade 2–3 was 3.82-fold lower than that in Grade 0 (*p* <  0.001). SHBG mRNA expression levels were positively correlated with HNF4α mRNA (*r* = 0.773, *p* <  0.001; Fig. 3d). Further results revealed that intrahepatic TG concentrations had significantly negative correlations with SHBG (*r* = − 0.715, *p* <  0.001) and HNF4α (*r* = − 0.622, *p* <  0.001) mRNA expression levels in liver, as well as serum SHBG levels (*r* = − 0.673, *p* <  0.001) (Fig. 3e-g).

## Discussion

The results of our community-based study illustrated an inverse association between serum levels of SHBG and the prevalence of NAFLD. In this study, the NAFLD participants had much lower SHBG concentrations than the non-NAFLD participants, and serum SHBG levels tended to decrease significantly with the increase of NAFLD severity in both females and males. Furthermore, we found that hepatic SHBG mRNA expression in 32 liver biopsy samples decreased as the grade of hepatic steatosis elevated. Our data also suggested that both serum SHBG levels and liver SHBG and HNF4α expression were negatively correlated with hepatic triglycerides.

Past research on the relationships between SHBG and NAFLD has often been performed in patients with metabolic disorders [11–16], and the results showed that serum SHBG levels were lower in NAFLD patients with metabolic disorders than in those without NAFLD. However, in these patients, the SHBG levels were lower than normal, so it is hard to apply these results to healthy populations. Additionally, due to the small sample sizes of these studies, the results have been inconclusive [12, 17, 18]. The present results that low SHBG groups were associated with higher odds of NAFLD in middle-aged and elderly Chinese adults are in line with those from a previous study reported by Wang N et al. [21]. However, the prevalence of diabetes, serum TG and BMI levels in their study were all much higher than that in our community population. Furthermore, the authors did not perform the subgroup analysis to exclude metabolic disorders, therefore limiting the conclusions from their study. Our study was based on a large, community-based, middle-aged and elderly Chinese population, after multivariate fully adjusted, participants in the highest quartile of SHBG compared with those in the lowest quartile of SHBG had a significantly lower prevalence of NAFLD (OR, 0.19, 95% CI, 0.13–0.27 in females; OR, 0.42, 95% CI, 0.23–0.77 in males; both *p* <  0.05). Research has shown a correlation between NAFLD and BMI, as the prevalence of NAFLD was reportedly lower in individuals with normal BMI levels and without metabolic risk factors [36]. NAFLD was also shown to have an obvious association with insulin resistance, which is a key risk factor for the development of type 2 diabetes mellitus [37]. Therefore, to estimate the association between SHBG and the risk of NAFLD without the confounding influences of overweight and insulin resistance, we further performed the subgroup analysis after stratifying the whole population by BMI and HOMA-IR status. Inverse associations between SHBG and the presence of NAFLD were still observed regardless of BMI and HOMA-IR status.

Two potential reasons may explain the association between SHBG and NAFLD. First, the occurrence and progression of the NAFLD may downregulate the hepatic expression of SHBG. Previous studies reported that both the accumulation of hepatic lipids [28] and chronic inflammation (pathogenesis of NAFLD) [38–40] could decrease the expression of SHBG. Other metabolic disorders concomitant with NAFLD, such as hyperglycaemia might also directly decrease the expression of SHBG [41]. Second, SHBG itself might contribute to the development of metabolic disorders. Ding EL et al. found that low circulating levels of SHBG played a strong causal role in the occurrence of type 2 diabetes in mendelian randomization analyses [42]. Moreover, SHBG overexpression in C57BL/ksJ-db/db mice reduced body weight gain [43], and SHBG overexpression in mouse models of NAFLD could significantly reduce liver fat accumulation by reducing key lipogenic enzymes [44].

As we know, liver tissue biopsy is the gold standard for diagnosing NAFLD and determining the grade of steatosis. To the best of our knowledge, our study was the first to investigate the relationship between SHBG and NAFLD in Asian biopsy-proven patients, although not in a large sample size. Our results revealed that HNF4α and SHBG mRNAs were significantly lower in subjects with high grades of hepatic steatosis, in accordance with a significantly decreasing trend in serum SHBG levels along with the elevated severity in our NAFLD subjects diagnosed by ultrasonography. Moreover, hepatic expression of HNF4α mRNA was positively correlated with SHBG mRNA. There were significantly inverse linear relationships between hepatic triglyceride concentrations and the levels of both SHBG and HNF4α mRNA expression levels. Serum SHBG levels were lower in subjects with high concentrations of hepatic triglycerides. Our results reveal a potential mechanism in that the accumulation of lipid in human liver is an important influencing factor of SHBG gene expression, thereby circulating serum levels of SHBG, and these effects are perhaps mediated through changes in nuclear transcription factor HNF4α. These results further supported the findings in our community-based study that serum SHBG is associated with NAFLD risk. Additionally, as the concentration of serum SHBG significantly decreased with the elevated degree of liver steatosis and injury, it can potentially be used to monitor the clinical response to interventions in the management of patients with NAFLD.

The prevalence of NAFLD in this middle-aged and elderly population, as diagnosed by ultrasound, was estimated to be 50.8%. We hypothesize that the following reasons can account for the high prevalence. First, the prevalence of NAFLD increases with age [45], and the average age of the participants in our community-based study was 60.6 years. Second, there may be selection bias in this research, as people with underlying health problems were more willing to participate in our study. Third, although the overall pooled prevalence of NAFLD was 20.09% in China [3], the epidemiology of NAFLD is greatly shaped by tremendous differences in lifestyle and economic growth between urban and rural areas within the same country. The relatively high prevalence of NAFLD in urbanized Asian areas including China has been reported [46–48], which was consistent with our results.

There were several limitations of this study, including a relatively limited biopsy-proven sample size. The diagnosis of NAFLD was based on ultrasonography, which is non-invasive, low cost, accessible, and widely used in large epidemiological studies, despite not being the gold standard as liver biopsy. Female subjects in the present study were more than double the proportion of males, and in order to avoid the potential bias, we conducted subgroup analysis after stratifying the whole population by sex. The results of logistic regression analysis showed that the associations between SHBG and the risk of NAFLD were consistent in females and males. Finally, this was a cross-sectional study design, which precluded us from establishing causal relationship. Further prospective studies are needed to evaluate the temporal relationship between SHBG and hepatic damage or predict how changes in SHBG will affect the progression of NAFLD.

## Conclusions

In summary, we found an inverse association between serum SHBG levels and the presence of NAFLD after multivariable adjustment for metabolic risk factors in community-dwelling participants. Research on liver specimens showed that not only did serum concentrations of SHBG decrease as the degree of hepatic steatosis exacerbated in NAFLD patients but also the liver mRNA expression of SHBG was negatively correlated with the severity of liver injury and intrahepatic TG. Whether SHBG can be selected as a noninvasive biomarker for NAFLD will be determined in future prospective studies and clinical trials.

## Additional files

Additional file 1: **Table S1.** Demographic characteristics, physical and metabolic measurements by sex and NAFLD status. (DOC 72 kb)

Additional file 2: **Table S2.** Association of serum SHBG levels with metabolic risk factors using multiple linear regression analysis. **Table S3**. Anthropometric and clinical parameters of control subjects and patients with NAFLD. (DOC 50 kb)

## Abbreviations

ALT: Alanine aminotransferase
ANCOVA: Analysis of covariance
AST: Aspartate aminotransferase
BMI: Body mass index
CI: Confidence interval
DHEAS: Dehydroepiandrosterone sulphate
HDL-C: High-density lipoprotein cholesterol
HNF4α: Hepatocyte nuclear factor 4-α
HOMA-IR: Homeostasis model assessment of insulin resistance
LDL-C: Low-density lipoprotein cholesterol
MET: Metabolic equivalent
NAFLD: Nonalcoholic fatty liver disease
OR: Odds ratio
SHBG: Sex hormone-binding globulin
TC: Total cholesterol
Testo: Testosterone
TG: Triglycerides
UA: Uric acid
WHR: Waist-to-hip ratio
