# Supplementary material for: Association of sex hormone-binding globulin with nonalcoholic fatty liver disease in Chinese adults
Source: Nutr Metab (Lond). 2018 Nov 8;15:79. doi: 10.1186/s12986-018-0313-8 (PMC6225668; doi:10.1186/s12986-018-0313-8)
Supplement: Supplementary file 1 — Table S1. Demographic characteristics, physical and metabolic measurements by sex and NAFLD status. (DOC 72 kb) [file 12986_2018_313_MOESM1_ESM.doc]

**Table S1.** Demographic characteristics, physical and metabolic measurements by sex and NAFLD status

|  | **Females** | | | **Males** | | | |  | **Total** |  |
| --- | --- | --- | --- | --- | --- | --- | --- | --- | --- | --- |
|  | non-NAFLD | NAFLD | *p* | | non-NAFLD | NAFLD | *p* | non-NAFLD | NAFLD | *p* |
| N | 1007 | 1002 |  | | 425 | 478 |  | 1432 | 1480 |  |
| Age (years) | 59.4 ± 5.4 | 60.2 ± 5.3 | 0.001 | | 63.1 ± 6.6 | 62.0 ± 6.0 | 0.010 | 60.5 ± 6.0 | 60.8 ± 5.6 | 0.220 |
| Postmenopausal status | 976 (96.9%) | 993 (99.1%) | 0.001 | | - | - | - | - | - | - |
| Household income, yuan/month/person | |  | 0.173 | |  |  | 0.295 |  |  | 0.084 |
| < 3000 | 794 (78.8%) | 764 (76.2%) |  | | 336 (79.1%) | 357 (74.7%) |  | 1130 (78.9%) | 1121 (75.8%) |  |
| 3000-6000 | 141 (14.0%) | 144 (14.4%) |  | | 60 (14.1%) | 83 (17.4%) |  | 201 (14.0%) | 227 (15.3%) |  |
| > 6000 | 72 (7.2%) | 94 (9.4%) |  | | 29 (6.8%) | 38 (7.9%) |  | 101 (7.1%) | 132 (8.9%) |  |
| Current smoking | 5 (0.5%) | 5 (0.5%) | 1.00 | | 148 (34.8%) | 171 (35.8%) | 0.781 | 153 (10.7%) | 176 (11.9%) | 0.320 |
| Current drinking | 39 (3.9%) | 39 (3.9%) | 1.00 | | 71 (16.7%) | 76 (15.9%) | 0.787 | 110 (7.7%) | 115 (7.8%) | 0.945 |
| Physical activity, MET/d | 34.5 ± 5.6 | 33.8 ± 5.2 | 0.007 | | 34.4 ± 6.1 | 33.4 ± 6.1 | 0.019 | 34.5 ± 5.7 | 33.7 ± 5.5 | < 0.001 |
| BMI (kg/m^2^) | 22.0 ± 2.6 | 24.8 ± 3.0 | < 0.001 | | 22.4 ± 2.4 | 25.3 ± 2.9 | < 0.001 | 22.1 ± 2.6 | 25.0 ± 3.0 | < 0.001 |
| WHR | 0.90 ± 0.07 | 0.93 ± 0.07 | < 0.001 | | 0.91 ± 0.06 | 0.95 ± 0.05 | < 0.001 | 0.90 ± 0.07 | 0.94 ± 0.06 | < 0.001 |
| Trunk fat percentage (%) | 34.2 ± 5.5 | 38.5 ± 4.1 | < 0.001 | | 24.9 ± 5.4 | 29.9 ± 4.3 | < 0.001 | 31.4 ± 6.9 | 35.7 ± 5.8 | < 0.001 |
| Hypertension | 192 (19.1%) | 361 (36.0%) | < 0.001 | | 125 (29.4%) | 174 (36.4%) | 0.028 | 317 (22.1%) | 535 (36.1%) | < 0.001 |
| Diabetes | 57 (5.7%) | 84 (8.4%) | 0.018 | | 36 (8.5%) | 51 (10.7%) | 0.309 | 93 (6.5%) | 135 (9.1%) | 0.009 |
| ALT (U/L) | 14 [11,18] | 16 [12,22] | < 0.001 | | 15 [12,20] | 19 [14,25] | < 0.001 | 14 [11,18] | 17 [13,23] | < 0.001 |
| AST (U/L) | 19 [16,22] | 18 [15,22] | 0.165 | | 19 [16,22] | 19 [16,22] | 0.556 | 19 [16,22] | 19 [16,22] | 0.427 |
| Glucose (mmol/L) | 4.71 [4.31,5.18] | 4.82 [4.40,5.38] | < 0.001 | | 4.83 [4.39,5.35] | 4.91 [4.50,5.49] | 0.020 | 4.75 [4.34,5.23] | 4.85 [4.43,5.41] | < 0.001 |
| HOMA-IR | 1.35 [0.94,1.89] | 2.21 [1.50,3.24] | < 0.001 | | 1.16 [0.85,1.76] | 2.09 [1.45,3.04] | < 0.001 | 1.31 [0.91,1.84] | 2.17 [1.49,3.18] | < 0.001 |
| TG (mmol/L) | 1.05 [0.81,1.47] | 1.45 [1.05,2.06] | < 0.001 | | 1.14 [0.81,1.54] | 1.44 [1.03,2.08] | < 0.001 | 1.08 [0.81,1.49] | 1.44 [1.04,2.06] | <0.001 |
| TC (mmol/L) | 5.73 ± 1.03 | 5.70 ± 1.03 | 0.476 | | 5.36 ± 1.04 | 5.24 ± 0.98 | 0.069 | 5.62 ± 1.05 | 5.55 ± 1.04 | 0.064 |
| HDL-C (mmol/L) | 1.63 ± 0.42 | 1.39 ± 0.36 | < 0.001 | | 1.38 ± 0.36 | 1.18 ± 0.31 | < 0.001 | 1.56 ± 0.42 | 1.32 ± 0.36 | < 0.001 |
| LDL-C (mmol/L) | 3.63 ± 0.90 | 3.71 ± 0.89 | 0.039 | | 3.42 ± 0.88 | 3.40 ± 0.93 | 0.831 | 3.57 ± 0.90 | 3.61 ± 0.91 | 0.169 |
| UA (μmol/L) | 314.5 ± 70.6 | 346.5 ± 76.9 | < 0.001 | | 388.9 ± 87.1 | 403.8 ± 85.5 | 0.009 | 336.6 ± 83.1 | 365.0 ± 84.1 | < 0.001 |
| SHBG (nmol/L) | 67.9 [50.0,87.8] | 45.6 [34.3,60.1] | < 0.001 | | 54.8 [42.7,70.4] | 40.6 [31.6,52.6] | < 0.001 | 63.4 [47.6,83.1] | 43.8 [33.4,56.8] | < 0.001 |
| Testo (ng/dL) | 25.8 [20.0,31.0] | 27.0 [22.1,34.0] | < 0.001 | | 692.0 [531.3,863.1] | 543.4 [420.7,704.2] | < 0.001 | 30.0 [22.1,470.9] | 33.2 [24.7,408.8] | 0.016 |
| DHEAS (μg/dL) | 98.7 [69.6,139.3] | 101.3 [69.3,141.3] | 0.497 | | 167.9 [122.1,219.4] | 169.6 [126.0,228.1] | 0.250 | 114.9 [79.5,165.4] | 119.5 [80.8,171.5] | 0.078 |

Data are presented as count (%) for categorical variables, mean (standard deviation) for non-skewed continuous variables, and median [25th, 75th percentiles] for skewed variables, with *p* values for χ^2^ tests, t-tests, and rank sum tests, respectively.

NAFLD, nonalcoholic fatty liver disease; MET, metabolic equivalent; BMI, body mass index; WHR, waist-to-hip ratio; ALT, alanine aminotransferase; AST, aspartate aminotransferase; HOMA-IR, homeostasis model assessment of insulin resistance; TG, triglycerides; TC, total cholesterol; HDL-C, high-density lipoprotein cholesterol; LDL-C, low-density lipoprotein cholesterol; UA, uric acid; SHBG, sex hormone-binding globulin; Testo, testosterone; DHEAS, dehydroepiandrosterone sulphate.
