# Supplementary material for: Association of sex hormone-binding globulin with nonalcoholic fatty liver disease in Chinese adults
Source: Nutr Metab (Lond). 2018 Nov 8;15:79. doi: 10.1186/s12986-018-0313-8 (PMC6225668; doi:10.1186/s12986-018-0313-8)
Supplement: Supplementary file 2 — Table S2. Association of serum SHBG levels with metabolic risk factors using multiple linear regression analysis. Table S3. Anthropometric and clinical parameters of control subjects and patients with NAFLD. (DOC 50 kb) [file 12986_2018_313_MOESM2_ESM.doc]

**Table S2. Association of serum SHBG levels with metabolic risk factors using multiple linear regression analysis**

|  | Unadjusted | | Adjusted | | |
| --- | --- | --- | --- | --- | --- |
| Variables | β ± SE | *p* | | β ± SE | *p* |
| BMI (kg/m^2^) | -0.028 ± 0.001 | < 0.001 | | -0.027 ± 0.001 | < 0.001 |
| WHR | -0.983 ± 0.052 | < 0.001 | | -0.952 ± 0.052 | < 0.001 |
| Trunk fat percentage (%) | -0.007 ± 0.001 | < 0.001 | | -0.015 ± 0.001 | < 0.001 |
| ALT (U/L) | -0.179 ± 0.018 | < 0.001 | | -0.141 ± 0.017 | < 0.001 |
| Glucose (mmol/L) | -0.553 ± 0.043 | < 0.001 | | -0.498 ± 0.047 | < 0.001 |
| HOMA-IR | -0.337 ± 0.012 | < 0.001 | | -0.334 ± 0.012 | < 0.001 |
| Triglyceride (mmol/L) | -0.320 ± 0.015 | < 0.001 | | -0.310 ± 0.014 | < 0.001 |
| Cholesterol (mmol/L) | 0.010 ± 0.003 | 0.003 | | 0.000 ± 0.003 | 0.998 |
| HDL-C (mmol/L) | 0.192 ± 0.008 | < 0.001 | | 0.175 ± 0.008 | < 0.001 |
| LDL-C (mmol/L) | 0.004 ± 0.004 | 0.332 | | -0.006 ± 0.004 | 0.152 |
| UA (mmol/L) | -0.780 ± 0.041 | < 0.001 | | -0.706 ± 0.043 | < 0.001 |

SHBG, ALT, fasting glucose, HOMA-IR and TG were put into linear regression after log-transformation. All variables were adjusted for age, sex, current smoking and drinking, physical activity (MET), hypertension and diabetes.

SHBG, sex hormone-binding globulin; BMI, body mass index; WHR, waist-to-hip ratio; ALT, alanine aminotransferase; HOMA-IR, homeostasis model assessment of insulin resistance; HDL-C, high-density lipoprotein cholesterol; LDL-C, low-density lipoprotein cholesterol; UA, uric acid.

**Table S3.** Anthropometric and clinical parameters of control subjects and patients with NAFLD

|  | non-NAFLD | NAFLD | *p* |
| --- | --- | --- | --- |
| N | 10 | 22 |  |
| Age (years) | 56.1 ± 8.5 | 59.9 ± 10.3 | 0.317 |
| Female/male | 4/6 | 11/11 | 0.712 |
| BMI (kg/m^2^) | 21.8 ± 3.5 | 23.6 ± 4.7 | 0.289 |
| Fasting glucose (mmol/L) | 4.66 ± 0.94 | 5.27 ± 0.96 | 0.106 |
| Fasting insulin (μU/mL) | 6.1 ± 2.8 | 10.0 ± 4.8 | 0.026 |
| HOMA-IR | 1.26 ± 0.47 | 2.34 ± 1.19 | 0.010 |
| Total cholesterol (mmol/L) | 5.41 ± 0.66 | 5.31 ± 0.77 | 0.730 |
| Triglyceride (mmol/L) | 1.04 ± 0.29 | 1.48 ± 0.39 | 0.004 |
| HDL-C (mmol/L) | 1.59 ± 0.25 | 1.26 ± 0.34 | 0.010 |
| LDL-C (mmol/L) | 3.32 ± 0.47 | 3.53 ± 0.67 | 0.360 |

Data are presented as mean (standard deviation) with *p* values for χ^2^ tests and t-tests.

NAFLD, nonalcoholic fatty liver disease; BMI, body mass index; HOMA-IR, homeostasis model assessment of insulin resistance; HDL-C, high-density lipoprotein cholesterol; LDL-C, low-density lipoprotein cholesterol.
